# Does Catheter Ablation Lower the Long-Term Risk of Stroke and Mortality in Patients with Atrial Fibrillation? A Concise Review of the Current State of Knowledge

**DOI:** 10.7759/cureus.9701

**Published:** 2020-08-12

**Authors:** Robert Ryad, Suhail M Saad-Omer, Farah Khan, Therese Limbana, Nusrat Jahan

**Affiliations:** 1 Internal Medicine, California Institute of Behavioural Neurosciences and Psychology, Brentwood, USA; 2 Medicine, California Institute of Behavioural Neurosciences and Psychology, Fairfield, USA; 3 Psychiatry, California Institute of Behavioural Neurosciences and Psychology, Fairfield, USA; 4 Internal Medicine, California Institute of Behavioural Neurosciences and Psychology, Fairfield, USA

**Keywords:** atrial fibrillation, catheter ablation, mortality, stroke

## Abstract

Atrial fibrillation (AF) is the most common cardiac arrhythmia worldwide and carries a significant risk of morbidity and mortality. Multiple trials have highlighted the benefit of catheter ablation over medical therapy in restoring sinus rhythm and improving quality of life. Whether it reduces long-term risk of stroke and mortality is still unclear.

We performed a literature search using the PubMed database to review the current state of knowledge regarding the long-term outcomes of stroke and mortality in ablated patients compared to patients who receive medical therapy. Our review mainly consisted of recent randomized controlled trials and large observational studies.

Results from large observational studies show that catheter ablation significantly reduces the risk of stroke in high-risk patients and mortality compared to medical therapy. However, randomized controlled trials have only demonstrated a mortality benefit in patients with comorbid systolic heart failure. In patients with paroxysmal AF, ablation therapy significantly limits the progression to persistent AF and has a higher efficacy in restoring sinus rhythm. Maintenance of sinus rhythm is the most important factor associated with lower long term risk of stroke and mortality.

Large randomized controlled trials similar to the Catheter Ablation Versus Anti-arrhythmic Drug Therapy for Atrial Fibrillation (CABANA) trial are still needed to clarify whether catheter ablation is superior over medical therapy in improving the long-term outcomes of stroke and mortality.

## Introduction and background

Atrial fibrillation (AF) is the most common cardiac arrhythmia and is regarded as a future epidemic among aging populations with an estimated worldwide prevalence of 0.51% and consistently increasing incidence rates [1]. It imposes a five-fold increased risk of stroke and a two-fold increased risk of dementia and mortality [2,3]. From another perspective, atrial fibrillation receives an annual share of 26 billion dollars of the US healthcare expenditure, and the increasing rate of incidence predicts further cost burden in the future [2].

Treatment for atrial fibrillation is either pharmacological or non-pharmacological. As for pharmacological therapy, the Atrial Fibrillation Follow-up Investigation of Rhythm Management (AFFIRM) trial was set to settle the long-standing debate of rate control vs. rhythm control and concluded that there was no superiority to either strategy. However, the investigators reported more adverse effects in the rhythm control arm, which could have potentially masked the results that failed to show any benefit (mortality or stroke risk reduction) for maintaining sinus rhythm [4].

Catheter ablation is the non-pharmacological form of therapy that has gained popularity in recent years. Randomized controlled trials have shown that in comparison to anti-arrhythmic drugs, catheter ablation is more successful in reducing arrhythmia recurrence and improving quality of life [5-8]. However, data comparing the long-term outcomes of stroke and mortality is still insufficient [9]. In this article, we review the current evidence to highlight whether there is any long-term benefit of catheter ablation over anti-arrhythmic drug therapy.

## Review

The literature search (Table 1) was performed on the PubMed database using regular keywords. Studies were then filtered based on the following inclusion and exclusion criteria.

Inclusion Criteria:

1) Studies on human subjects written in English

2) Studies and articles published between the years 2000-2020

3) Full text available

4) Study subjects > 18 years old with atrial fibrillation

Exclusion Criteria:

1) Case-reports, case-series, systematic Reviews and meta-analyses

2) Articles irrelevant to the study objective

**Table 1:** Cumulative search results before and after applying the inclusion criteria.

Search buildup	Total Results
Regular keywords- Atrial Fibrillation Catheter ablation mortality and stroke risk	286
Inclusion Criteria	
Published between 2000-2020	284
English language	270
Human Species	232
Full-text articles	224

**Figure 1:**
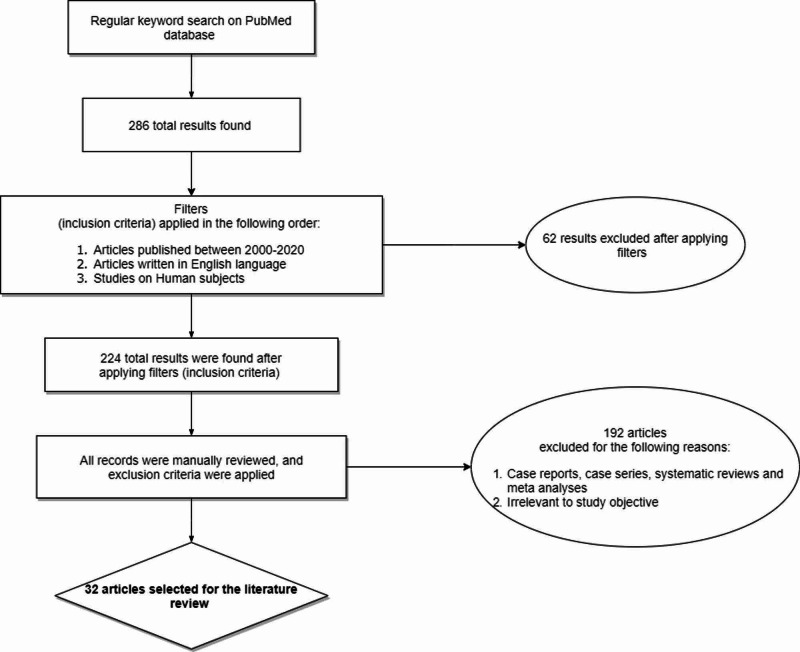
Flowchart demonstrating the literature search process.

After applying the inclusion criteria, search results (224 in total) were then manually reviewed to apply exclusion criteria, yielding 32 relevant articles that did not include case reports, case series, systematic reviews, and meta-analyses. Of the 32 articles, 16 were observational studies (mostly retrospective cohort), five were review articles, and 11 were randomized controlled trials. In addition, the American Heart Association (AHA)/American College of Cardiology (ACC) 2014 and 2019 guidelines were also reviewed to shed light on current treatment guidelines. Total study subjects of clinical trials were 5,542 compared to 98,445 patients who were enrolled in observational studies. Twenty-seven of the 32 chosen articles have been published within the last six years (84.375%). The review encompasses catheter ablation in AF patients with and without heart failure (HF). Figure 1 summarizes the literature search process.

In the analysis of selected articles for the presented literature, we found sufficient evidence of the long-term benefit of catheter ablation in specific sub-populations of AF patients. However, evidence on whether catheter ablation reduces mortality and stroke risk in all patients with atrial fibrillation is still lacking. Articles reviewed specifically point to mortality benefit in patients with AF and HF [10-12], along with stroke risk reduction in patients with AF and high CHA₂DS₂-VASc score (≥2) [13-15]. Multiple studies have also highlighted that maintaining sinus rhythm was the most important factor in predicting stroke-free survival and lower mortality [15-17]. Furthermore, besides the current consensus of preserving catheter ablation for symptomatic patients with drug-refractory atrial fibrillation [18], there is sufficient evidence that catheter ablation for patients with paroxysmal AF is highly effective in curtailing progression to persistent AF [19] and achieves better results of sinus rhythm maintenance [20]. Observational studies showing a mortality benefit with catheter ablation are summarized in Table 2.

**Table 2:** Studies that demonstrate the long-term mortality benefit of catheter ablation. HR: Hazard Ratio, AF: atrial fibrillation

Author Name/ Year of publication	Study Design	Study Subjects	Main findings
Arai et al., 2019 [21]	Propensity-matched retrospective observational study	872 patients from Tokyo registry data	Significantly lower rate of all-cause death in the ablation group compared to the non-ablation group (HR= 0.37, 95% Confidence Interval: 0.12-0.93, p=0.041)
Srivatsa et al., 2018 [22]	Propensity-matched retrospective observational study	8,338 multi-ethnic patients in California	Lower 30-day to 5-year mortality of 0.9% (person-years) in the ablation group vs 2% in the no-ablation control group; HR=0.57 (95% Confidence Interval: 0.43-0.74, p<0.0001)
Jarman et al., 2018 [23]	Propensity-matched retrospective cohort study	1,116 patients in England	50% lower risk of mortality in the ablation group compared to the anti-arrhythmic drug group (p= 0.0082) over 3 years of follow-up.
Friberg et al., 2016 [14]	Propensity-matched retrospective observational study	4,992 patients from Swedish health registries	The annual mortality rate was 0.77% in the ablated group compared to 1.62% in the non-ablated group (p<0.001), with cardio-vascular deaths being twice as common in the non-ablated group compared to the ablated group.
Nademanee et al., 2015 [17]	Retrospective cohort study	324 elderly patients (≥ 75 years old) with symptomatic AF	Significantly lower 5-year mortality in ablation patients who successfully maintained normal sinus rhythm compared to ablation patients who failed to maintain sinus rhythm and patients assigned to medical rate control (87%, 52%, and 42% respectively, p<0.0001). Catheter ablation was more successful in reducing AF burden and maintaining normal sinus rhythm, which was an independent factor of better survival.
Lin et al., 2013 [15]	Propensity matched retrospective observational study	348 patients with symptomatic AF and CHA₂DS₂-VASc score ≥ 1	Lower rate of all-cause mortality and cardiovascular deaths in ablated group vs non-ablated group (2.95% vs. 0.74% per year; p < 0.01 and 1.77% vs. 0% per year; p = 0.001 respectively) over a mean follow-up period of 47 ± 23 months.
Bunch et al., 2011 [24]	Prospective cohort study	37,908 predominantly white patients	5-year mortality rates for ablated AF patients was significantly lower than non-ablated AF patients and similar to non-AF patients (7.6%, 27.9%. and 11.4% respectively)

Most of the studies have attributed possible long-term mortality benefit of catheter ablation to the higher efficacy in restoring sinus rhythm and reducing AF recurrence despite often required repeat procedures [5, 17, 20, 25]. On the other hand, some studies have shown no mortality benefit of catheter ablation over medical therapy, including the long-awaited, large, randomized, controlled Catheter Ablation Versus Anti-arrhythmic Drug Therapy for Atrial Fibrillation (CABANA) trial. In this multi-center, open-label study, 1108 patients were assigned to receive catheter ablation against 1096 matched controls receiving medical therapy. The main findings of the study were that over a median follow-up period of 48.5 months; there was no significant difference in all-cause mortality (5.2% in ablation group vs. 6.1% in drug therapy group, hazard ratio [HR]: 0.85 [95% CI, 0.60-1.21]; p=0.38) between both groups using intention-to-treat analysis despite ablation therapy showing better results in reducing AF recurrence (49.9% in ablation group vs. 69.5% in drug therapy group, HR: 0.52 [95% CI, 0.45-0.60]; p<.001). However, treatment assignment sensitivity analysis showed that there is a benefit for ablation over medical therapy if patients assigned to ablative therapy did receive ablation. Moreover, there was a 27% crossover from the medical therapy group to the ablation group, which highlights the relative inefficacy and hazardous side effect profile of anti-arrhythmic medications. In a recent, large 2019 propensity-matched observational study it was also concluded that there was no difference in mortality and cardiovascular deaths between ablated patients and medically treated patients [26]. The study follow-up period, however, was only one year, which may indicate that the mortality benefits of ablation therapy are not seen before ≥ two years of post-ablation follow-up.

AF and heart failure are frequently co-existent conditions. It is often difficult for clinicians to determine the ‘chicken-egg’ relationship between the two as both conditions share similar risk factors and may lead to each other. Atrial fibrillation compromises left ventricular function due to loss of atrial systole and may cause tachycardia-mediated cardiomyopathy [27], while heart failure may induce atrial remodeling and fibrosis, which promotes AF. Multiple studies (Table 3), including randomized controlled trials, have demonstrated significant mortality benefit, improved left ventricular function, and quality of life with catheter ablation in patients with AF and comorbid heart failure.

**Table 3:** Studies which show mortality benefit and LVEF improvement in HF patients who receive catheter ablation. NYHA: New York Heart Association, LVEF: Left Ventricular Ejection Fraction, HR: Hazard Ratio, CI: Confidence Interval, AF: atrial fibrillation

Author Name/Year of publication	Study Design	Study Subjects	Main findings
Marrouche et al., 2018 [11]	Multi-center, open-label, randomized, controlled trial	363 patients with paroxysmal or persistent atrial fibrillation, NYHA class ≥ II, LVEF ≤ 35%, and an implanted defibrillator.	Mortality rate was 13.4% in ablation group compared to 25% in medical therapy group (HR=0.53; 95% CI, 0.32-0.86; p=0.01). More specifically, cardiovascular deaths were almost twice more common in the medical-therapy group than the ablation group (22.3% vs 11.1%; 95% CI, 0.29-0.84; p=0.009).
Geng et al., 2017 [28]	Multi-center, open-label, randomized, controlled trial	66 patients aged between 18-85 years, with persistent AF, EF ≤ 45%, NYHA class ≥ II, and no significant coronary artery disease.	At 6 months, LVEF normalized (≥50%) in 58% of the patients in the ablation group compared to 9% in the medical therapy (rate-control) group (p=0.0002). Absolute LVEF increased by 18 ± 13% in the ablation group compared with 4.4 ± 13% in the medical therapy group (p< 0.0001)
Luigi et al., 2016 [12]	Randomized, parallel-group, multicenter study	203 patients with persistent AF, cardiac defibrillator, NYHA Class II to III, and left ventricular ejection fraction <40%.	Significantly lower mortality was observed in the ablation group compared to the amiodarone-treated group (8% vs. 18%, p=0.037) over a minimum follow-up period of 24 months.
Bunch et al., 2015 [10]	Retrospective cohort study	2,403 patients with an ejection fraction ≤ 35% matched according to age and sex.	Long-term mortality rates were lower in the ablated AF group compared to AF with no ablation group and no AF group (27%, 55%, and 50%, respectively (p< 0.0001) and was attributed to lower cardiovascular mortality in ablated AF group.
Hunter et al., 2014 [29]	Single-center, randomized, controlled trial	50 patients with persistent AF, symptomatic HF(NYHA class ≥ II), LVEF < 50%.	At 6 months, LVEF in the ablation group was 40±12% compared with 31±13% in the rate control group (p=0.015), the NYHA score was 1.6 (CI: 1.4–1.9) in the ablation group compared with 2.4 (CI: 2.1–2.6) in the medical group (p<0.001) and Minnesota living with HF questionnaire score was 24±22 versus 47±22 in the rate control group (p=0.001).

In contrast to the trials mentioned above, the Atrial Fibrillation Management in Congestive Heart Failure With Ablation (AMICA) trial, a randomized controlled trial in 2019, failed to show a significant benefit of ablation therapy over medical therapy [30]. Unlike other trials, medical therapy improved left ventricular ejection fraction (LVEF) in patients with persistent AF and left ventricular systolic dysfunction by a magnitude not deemed statistically different from that of ablation therapy at one year (7.3% [CI: 4.3%-10.3%] and 8.8% [CI: 5.8%-11.9%], respectively). The study authors explained that the limited benefit of catheter ablation in their study subjects was because they were generally sicker and with more advanced HF compared with the patient population in other studies (particularly the Catheter Ablation vs. Standard Conventional Treatment in Patients With LV Dysfunction and AF [CASTLE-AF] trial). They concluded that ablation therapy might have limited benefit over medical treatment in improving left ventricular function and quality of life in patients with seriously advanced HF despite achieving lower AF burden. Overall, the evidence pointing towards the mortality benefit of ablation therapy in AF patients with comorbid systolic heart failure is quite compelling. In fact, the 2019 ACC/AHA guidelines for AF management mention that it is reasonable to consider ablation therapy in AF patients with reduced LVEF (Class of Recommendation: IIb, Level of Evidence: B-R) [31].

Regarding the long-term risk of stroke, in 2017, a large study in Israel investigated the long-term outcomes of ablation therapy in 969 patients against a propensity score-matched cohort of 3772 non-ablated patients (ratio of 1: ≤4). At baseline, most of the study subjects in both groups were at high risk for stroke (mean CHA₂DS₂-VASc score: 3.6 ± 2.0). However, after long-term follow-up, the rates of transient ischemic attack (TIA), stroke, and mortality were significantly lower among the ablation group compared with the non-ablation group [13]. Other previously discussed observational studies have reported similar findings among patients at high risk for stroke [14-16] Furthermore, in patients with a previous history of cerebrovascular accident, it was found that the risk of a subsequent stroke in ablated AF patients was similar to that of non-AF patients and significantly lower than that of non-ablated AF patients [32]. Despite the apparent benefit, the role of oral anticoagulants (OACs) in stroke prevention should not be overlooked in ablated patients. In a large recent study of US national registry data, it was found that in about one out of four patients treated with catheter ablation, OACs were discontinued despite having a high risk of stroke (CHA₂DS₂-VASc ≥ 2) [26]. A recent propensity-matched retrospective cohort study in Japan investigated the long-term risk of stroke in ablated patients with non-ablated patients. It was found that there was no difference in the incidence of stroke between ablated and non-ablated patients over a follow-up period of 28.0 ± 17.1 months despite significantly lower rates of mortality and cardiovascular deaths in the ablated group. Interestingly, OACs were discontinued in 230 out of 512 ablated patients, among which 25% had a CHA₂DS₂-VASc score >3 [21]. Thus, it remains clear that ablation therapy should not be used for the sake of discontinuing OACs in patients at high risk of stroke, but instead both therapies should be combined for better mitigation of stroke risk.

Some studies suggest a unique advantage for ablation therapy in patients with paroxysmal AF. In these patients, it has been found that catheter ablation limits the progression to persistent AF by a rate of <3% over two to five years compared to a rate of 11-26% over one year observed in medically treated patients [19]. Furthermore, in another study, it was found that patients with paroxysmal AF were more likely to maintain sinus rhythm after ablation therapy compared to those with persistent AF (proportion of time in sinus rhythm: 0.74 ±0.34 vs. 0.52±0.38; p<0.0001) [20]. The mortality rate in this study was significantly higher over 10 years in patients with persistent AF than those with paroxysmal AF and similar to other studies, maintenance of sinus rhythm was associated with a substantially lower risk of cardiac mortality (HR: 0.41; 95% CI:0.20-0.84, p=0.015). Predictors of mortality were age, comorbid diseases (coronary artery disease, hypertension, diabetes mellitus, and obstructive sleep apnea), lower LVEF, and increased left atrium (LA) size.

Discussing long-term outcomes of a procedure may seem futile if we do not consider the different modes of carrying it out and whether one method is more successful. In 2019, a randomized controlled trial on 346 patients with drug-refractory paroxysmal AF compared radiofrequency ablation with 2-minute cryoballoon ablation and 4-minute cryoballoon ablation. All patients were subjected to continuous rhythm monitoring using an implantable loop recorder to test the primary outcome of freedom from arrhythmia recurrence at one year. The efficacy of the three procedures at one year was not statistically different (79.1% in radiofrequency group vs. 78.2% in 4-minute cryoballoon ablation group, and 73.3% in the 2-minute cryoballoon ablation; p=0.26).

In general, catheter ablation is considered a relatively safe procedure with a low risk of complications. Results from the California Study of Ablation for Atrial Fibrillation (CAABL-AF) study that included 4169 ablated patients showed a 0.001% risk of mortality and 0.003% risk of stroke within 30 days of the procedure [22]. Another propensity-matched retrospective study compared the outcome of stroke in 12,177 ablated patients to an equal group of cardioversion patients and found out that the initial risk of stroke within 30 days of the procedure was higher in the ablation group (RR: 1.53; P=0.05) [33]. In contrast, a 2014 randomized controlled trial investigated the periprocedural (within 48 hours of ablation) risk of stroke in patients who discontinued warfarin before the procedure (bridged with heparin) versus patients who were maintained on warfarin and found that uninterrupted warfarin reduced the risk of thromboembolic events by 95% [34]. This finding surely carries a lot of weight in reducing peri-ablation stroke risk and shall modify the current standards of practice, especially if complemented by other similar trials.

The main limitation of this study is that we did not include data from systematic reviews and meta-analyses. However, most of the studies reviewed were recent large observational studies or randomized controlled trials to shed light on the latest state of knowledge.

## Conclusions

Several observational studies have found that catheter ablation for atrial fibrillation reduces long-term risk of stroke and mortality compared to medical therapy. Randomized controlled trials have mainly demonstrated this benefit in the HF population but not the non-HF population. Reduction in stroke risk was most significant among patients with a high CHA₂DS₂-VASc score (≥2) and those with a previous history of stroke, provided anti-coagulant therapy is not discontinued. The most important factor implicated in the reduction of stroke and mortality is maintenance of sinus rhythm, which is better achieved by ablation therapy compared to anti-arrhythmic medications. Catheter ablation may offer a special advantage in patients with paroxysmal AF since it has been shown to better limit its progression to persistent AF than anti-arrhythmic medication. Furthermore, patients with paroxysmal AF who undergo catheter ablation tend to maintain sinus rhythm for a larger proportion of time than persistent AF patients. Catheter ablation is considered a relatively safe procedure with minimal periprocedural stroke and mortality risk. Periprocedural stroke risk is significantly reduced when warfarin therapy is maintained as opposed to warfarin discontinuation and bridging with heparin before the procedure. Large randomized controlled trials similar to the CABANA trial are still needed to confirm the long-term benefit of catheter ablation over medical therapy, as shown by observational studies as findings of the CABANA trial have been inconclusive and showed no significant superiority.
